# Modeling of a Novel Coaxial Ducted Fan Aerial Robot Combined with Corner Environment by Using Artificial Neural Network

**DOI:** 10.3390/s20205805

**Published:** 2020-10-14

**Authors:** Tianfu Ai, Bin Xu, Changle Xiang, Wei Fan, Yibo Zhang

**Affiliations:** 1 School of Mechanical Engineering, Beijing Institute of Technology, Beijing 100081, China; aitianfu@bit.edu.cn (T.A.); xiangcl@bit.edu.cn (C.X.); fanweixx@bit.edu.cn (W.F.); yibo.zhang@bit.edu.cn (Y.Z.); 2 Beijing Institute of Technology Chongqing Innovation Center, Chongqing 401120, China

**Keywords:** corner effect, ducted fan, aerodynamic interference, artificial neural network

## Abstract

A novel coaxial ducted fan aerial robot with a manipulator is proposed which can achieve some hover operation tasks in a corner environment, such as switching on and off a wall-attached button on the corner. In order to study the aerodynamic interference between the prototype and the environment when the aerial robot is hovering in the corner environment, a method for the comprehensive modeling of the prototype and corner environment based on the artificial neural network is presented. By using the CFD simulation software, the flow field of the prototype at different positions with the corner effect is analyzed. After determining the input, output and structure of the neural network model, the Adam and gradient descent algorithms are selected as the neural network training algorithms, respectively. In addition, to optimize the initial weights and biases of the neural network model, the genetic algorithm is precisely used. The three-dimensional prediction surfaces generated by the three methods of the neural network, kriging surface and the polynomial fitting are compared. The results show that the neural network has high prediction accuracy, and can be applied to the comprehensive modeling of the prototype and the corner environment.

## 1. Introduction

Due to their strong maneuverability and high flexibility, unmanned aerial vehicles (UAVs) have gained great application prospects in many fields and scenarios in recent years. For example, UAVs have been used in communication relays, meteorological monitoring, disaster monitoring, and several other fields, which shows that UAVs perform different tasks with a variety of tools according to their type, function, operating characteristics and mission objectives, and liberate human hands in certain scenarios or confined spaces, and can achieve different operation requirements. The use scenarios of UAVs are no longer limited to simple open environments, and the transition to indoor and complex environments has become a trend. However, the performance of UAVs in complex environment is significantly different from that in open space. In complex environments, a UAV is constrained by complex and changeable boundaries such as buildings, trees and terrain, which affects its performance and operation state. In addition to the collision between UAVs and obstacles in complex environments, the biggest potential danger is the proximity effect, such as ground effect, wall effect, ceiling effect, etc. When the UAV’s rotor rotates, it will drive the surrounding airflow. Therefore, when the UAV approaches the target, the airflow between the rotor and the surrounding environment will interact, and affect the free diffusion of the airflow. This effect will be more prominent when the UAV is hovering and flying at a low speed.

**Figure 1:**
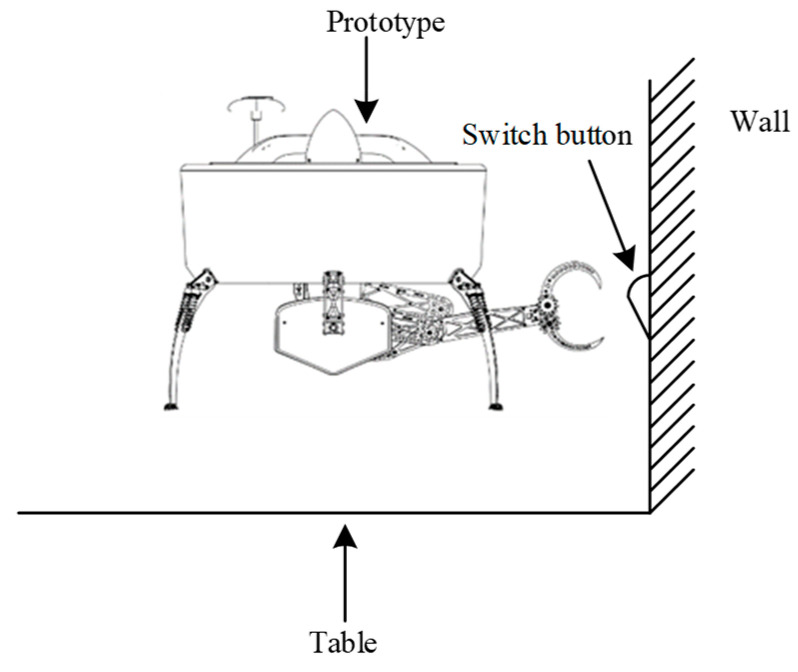
Schematic diagram of a typical corner effect scenario.

Most previous studies considered proximity effects, such as ground effect, wall effect, and ceiling effect, respectively. Each of the three effects only considers aerodynamic performance changes caused by the distance variation between the UAV and a single obstacle or flat surface. When a UAV is operating in a complex environment, there is often more than a single obstacle or aerodynamic interference between the UAV and the environment, generally combined with combinations of several effects. For example, the corner effect is obtained by the combination of the wall effect and the ground effect, and the channel effect is obtained by the combination of the ceiling effect and the ground effect, etc. Among the various combination effects, the corner effect is the most common and typical one, which can be encountered in many cases. In this paper, we define the corner environment to include a plane parallel to the ground and a plane perpendicular to the ground. For example, we use a UAV with a robotic arm to switch a wall-attached button on a table on and off. In this situation, the UAV needs to hover over the table and keep a certain distance from the wall. The UAV with the corner effect is shown in Figure 1.

Compared with UAVs with an open-rotor structure, the UAV with a ducted structure has better safety performance because of its external duct, which can avoid rotor contact with the outside directly. In addition, when the rotor blade size is the same, the thrust generated by the duct is greater than that of the open-rotor due to the air flow at the lip [1]. At the same time, when the size is the same, the ducted structure UAVs can carry greater loads than the open-rotor UAVs, making it possible to carry different tools for different operations. Therefore, a ducted structure UAV is more suitable than an open structure UAV in a complex environment.

However, due to the characteristics of the ducted structure, in complex environments, our prototype is generally used for low-speed flight and hovering operations near objects. At this time, due to the influence of the external environment, there will be a lot of aerodynamic interference, resulting in the prototype not being able to hover near the object, affecting the efficiency and accuracy of the operation. Therefore, it is necessary to make clear the aerodynamic effects of the interaction between the prototype and the environment when the UAV is in a complex environment. This is an unsteady aerodynamic problem. In the past, for such problems, the aerodynamic interference between the prototype and the environment in different scenarios were generally obtained through CFD [2,3,4] and wind tunnel experiments [5,6,7]. However, CFD simulation and wind tunnel experiments also have certain limitations. The main cost of CFD software simulation is time, which depends on the specific issues that need to be solved for the study. Usually, each more complicated CFD simulation takes a lot of time. Wind tunnel testing is an effective tool for collecting aerodynamic data. However, the cost of wind tunnel experiments is very high, and each wind tunnel experiment requires a lot of time and resources. In addition, through the above two methods, we can only obtain the aerodynamic characteristics of the prototype at some positions, while the aerodynamic characteristics at each position cannot be determined. 

At the same time, the operating efficiency of the prototype will be affected as the prototype is too far away from the target object. If it is too close to the target object, the prototype’s stability and safety will not be guaranteed. Therefore, it is necessary to clarify the aerodynamic interference caused by the change in the distance between the aircraft and different obstacles in the environment; that is, the aerodynamic characteristics at different positions. Some previous methods used a nonlinear disturbance observer [8,9] to estimate the aerodynamic interference between obstacles and the prototype in the environment, or used adaptive control strategies [10,11] to compensate for it. However, the nonlinear disturbance observer method often needs to clarify the dynamic characteristics brought by aerodynamics. In complex environments, aerodynamic characteristics are changeable and difficult to obtain. The adaptive control method is often time-consuming and computationally intensive, and the required controller cost is relatively high. When working in complex environments, prototypes are often expected to be as small as possible, which will limit the size and computing power of their embedded processors. Therefore, the above two methods are often computationally intensive and require powerful processors to perform calculations, which are not suitable for aircraft operating in complex environments.

Traditional theories such as the linear aerodynamic coefficient cannot simulate the behavior of a UAV in a complex environment. Therefore, a nonlinear aerodynamic model needs to be established to accurately predict the actual behavior of the UAV hovering in the corner environment. UAV system identification is a useful tool for nonlinear aerodynamic modeling. At present, mathematical tools used for aircraft system identification include polynomials [12,13], support vector machines [14,15,16], multivariate orthogonal functions [17], the Extended Kalman Filter method [18,19], and artificial neural networks [20,21,22,23], and many other mathematical tools are used for aircraft system identification.

In recent years, neural networks have proven to be a useful method that can estimate a wide range of functions and effectively model nonlinear aerodynamics regardless of the structure of the UAV [24,25,26]. Therefore, many researchers have used neural networks to study unsteady aerodynamics. Dmitry I.I. [27] used neural networks to model the aerodynamic characteristics of UAV at high attack angles, compared the modeling effects of feedforward neural networks and recurrent neural networks, and performed wind tunnel experiments on prototypes to verify the prediction accuracy of the neural network model. In order to describe the aerodynamic characteristics of flapping kinematics, Dilek Funda Kurtulus used an artificial neural network to model it [28]. The performance of the neural network is predicted by lift coefficient and drag coefficient in aerodynamics. Jiang Y. [29] presented a method for unsteady aerodynamic modeling by support vector machines and artificial neural. The experiment results show that deep learning can be applied to unsteady aerodynamic modeling. Kou J. et al. [30] presented a multi-kernel neural network and applied it to a nonlinear unsteady aerodynamic model in varying flow conditions. By comparison with the kernel RBF neural network model, a multi-kernel neural network can be more effectively used in the study of unsteady aerodynamics.

The artificial neural network is proposed to model the prototype and corner environment synthetically, which can predict the aerodynamic interference of the prototype hovering at different positions in the corner environment. Furthermore, the various aerodynamic interference between corner environment and the prototype under the corner effect can be clarified, which is also very helpful for the stability control of the UAV system under the corner effect in the next step. At the same time, it also helps to improve the space mobility and survivability of UAVs in complex environments such as corners, and to meet the urgent needs of related operations. 

This paper mainly has two contributions. One is to analyze the flow field and aerodynamic characteristics of a novel coaxial ducted fan aerial robot in corner environment. The other is to establish a comprehensive model taking both the corner environment and the prototype into consideration by using an artificial neural network. In addition, to optimize the neural network model, the genetic algorithm is precisely used, and two training algorithms, Adam and the gradient descent, are selected, respectively. Finally, the neural network model with and without the genetic algorithm and the neural network model with different training algorithms are compared, and the result shows that GA-ADAM-BP neural networks have better prediction accuracy. To our knowledge, our approach is the first to integratedly model the UAV and the environment.

This study aims to establish the unsteady aerodynamic characteristics considering the prototype and corner environment, and use a neural network to model them comprehensively. The contents of the remaining sections are as follows: Section 2 gives the aerodynamic influence of a ducted fan in the corner effect; Section 3 proposes using a neural network for comprehensive modeling of the prototype and corner environment; Section 4 gives the comparison between the neural network prediction model and other fitting methods; and the main conclusions and future outlook are presented in Section 5.

## 2. The Aerodynamic Influence of Corner Effect on the Prototype

**Figure 2:**
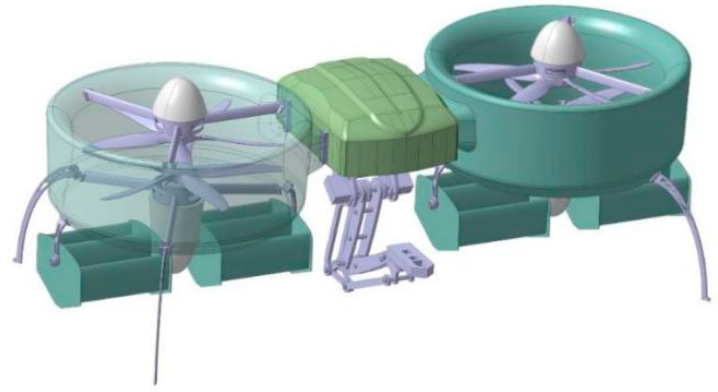
Novel prototype with manipulation.

In previous research, we have done a series of relevant studies on the prototype [31,32,33,34,35]. Through the continuous optimization of the prototype structure and design, the latest prototype with the manipulation is shown in Figure 2. The key parameters of the prototype are given in Table 1.

### 2.1. Analysis of Flow Field for Corner Effect

Computational fluid dynamic simulations can be used to gather qualitative information about the disturbance behaviors when the prototype is flying near obstacles. This information may be analyzed in the future with the help of artificial intelligent algorithms to acquire new knowledge or reduce computational time. In this paper, the CFD software that was used is Fluent. Fluent can be used in all fields related to fluids, heat transfer, and chemical reactions. When the prototype hovers in different positions in the corner environment, the application of Fluent software in this paper can not only analyze the flow field of the prototype but also obtain the aerodynamic performance of the prototype. According to the geometric model of the prototype, it is processed and meshed. Then the mesh is imported into Fluent, and the shear stress transport (SST) *k*-*w* turbulence model is applied in simulations. Next, we perform related operations on cell zone conditions, boundary conditions, and mesh interface, and set corresponding simulation parameters. When the output of the Fluent simulation is set to the three direction forces and moments of the prototype, the simulation starts to iterate. Once the iteration meets the requirements, the iteration is considered to have converged, and the corresponding data are output.

To the authors’ knowledge, there are few studies about the corner effect of open-rotor UAVs [36]. At the same time, the study of the corner effect on ducted-structure aerial robots is even rarer. It is shown in Figure 2 that the manipulation is installed in the middle area of the two ducts. Therefore, when the prototype is working, its two ducts need be parallel to the target object. 

When the prototype produces a wall effect, there is mutual interference between the duct, the rotors, the ground and the wall. It is necessary to analyze the flow field of the corner effect at different positions of the prototype. By analyzing the flow field of the prototype in the corner effect, the flow field changes caused by the position change of the prototype at different corner positions can be determined. In addition, the artificial neural network modeling of the prototype in the corner environment in the next step also requires a large number of unsteady data as a basis, which can also be obtained by analyzing the different positions of the prototype in the corner environment through CFD. Because the size of the manipulation tool is small relative to the whole prototype, and the aerodynamic influence in complex environment is quiet weak, so the manipulation tool is not considered when analyzing the flow field of the prototype under a corner effect in this section.

**Figure 3:**
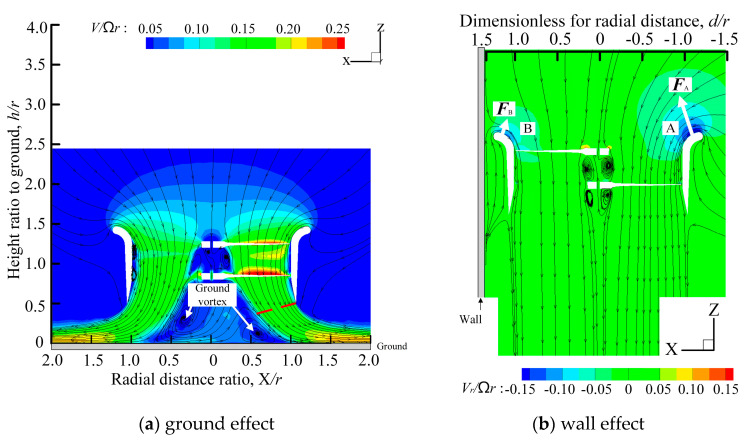
Velocity cloud of ducted fan with ground effect or wall effect.

Due to the symmetry of the prototype structure, a single ducted fan with a coaxial rotor was used instead of the prototype for analysis. Considering that the distance between the front ducted fan and the rear ducted fan is sufficiently large, the aerodynamic interference between them can be ignored. In an unconstrained environment, the prototype can hover at the speed of 4000 rpm, so the subsequent flow field analysis is based on the rotor speed of 4000 rpm. In order to better analyze the flow field of the prototype with corner effect at different positions, the flow field of the ducted fan is also analyzed when there are with the ground effect and the wall effect. The flow field analysis results are shown in the Figure 3.

Figure 3a shows that when the ducted fan operates under the ground effect, the airflow that diffuses outward from the duct interacts with the airflow that returns from the ground to produce a ground vortex under the duct. According to previous research [34], generally speaking, when the ducted fan is further from the ground, the degrees of ground vortexes will decrease, and when the ducted fan is closer to the ground, the degrees of ground vortexes will increase, which is not conducive to the stable hovering of the prototype at a given altitude. *h* is the distance from the lower surface of the upper paddle hub to the ground, *d* is the distance from the outer lip of the duct to the wall, X is the radial distance of the duct, and *r* is the rotor radius.

When the ducted fan operates with the wall effect, the wall prevents the gas movement, slowing down air flow at the duct lip B close to the wall, and its pressure rises, resulting in the imbalance in the total lift force, and the force at *F*_A_ is greater than that at *F*_B_, as shown in Figure 3b.

**Figure 4:**
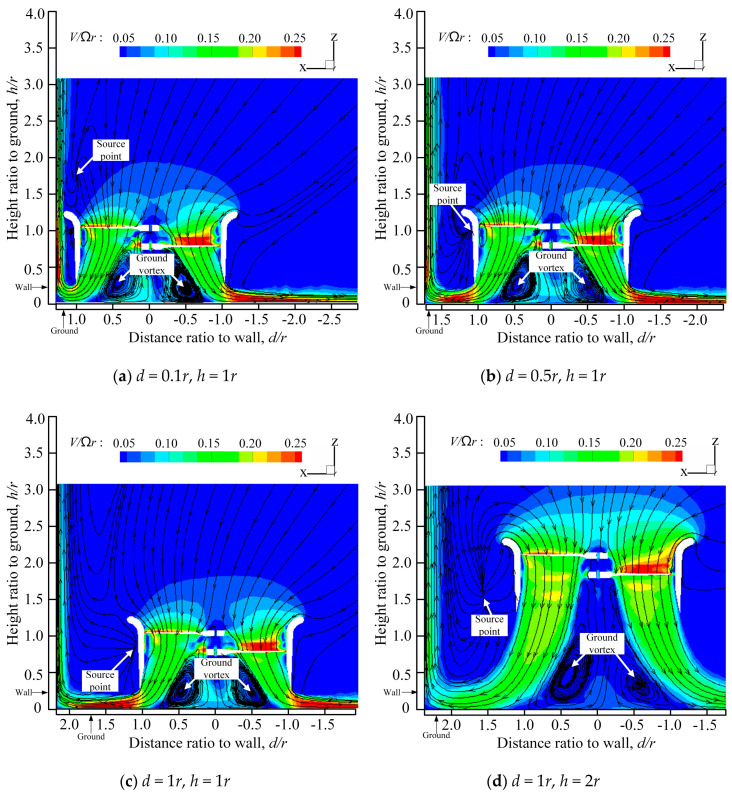

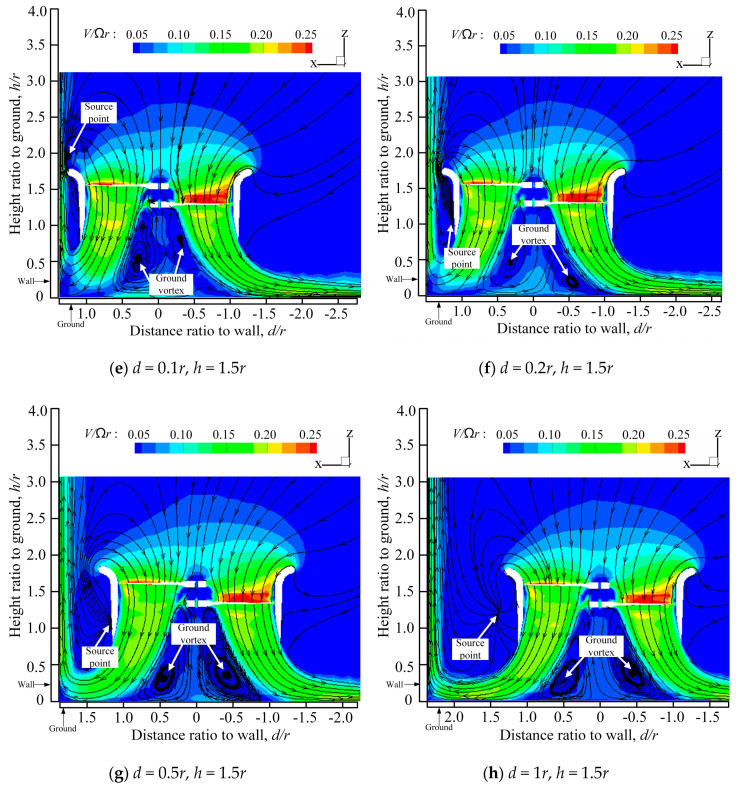
The velocity cloud graphs of a ducted fan at different positions with the corner effect.

The velocity cloud graphs of the ducted fan at different positions with the corner effect can be seen in Figure 4.

From Figure 4, we can observe the speed cloud diagram of the ducted fan at different positions under the corner effect. *h* is the distance from the lower surface of the upper paddle hub to the ground, *d* is the distance from the outer lip of the duct to the wall, and *r* is the rotor radius. During the outflow, the ground vortex is formed due to the obstruction of the ground. At the same time, due to the existence of the wall, a source point is formed outside the duct near the wall. 

When the distance between the duct and the ground is relatively large, it can be seen from Figure 4d–h that compared with Figure 3b, due to the existence of the ground, the airflow from the duct near the wall cannot diffuse downward freely, and will be refracted by the ground and rise along the wall. The part of the airflow at the source point interacts with the wall and rises along the wall, while the other part will rise above the duct and flow into the duct. With the decrease in the distance between the duct and the wall, the amount of airflow into the duct will increase.

When the distance between the duct and the ground is relatively small, it can be seen from Figure 4a,b that compared with Figure 4e,f, the degrees of the ground vortexes are intensified. At the same time, due to the obstruction of the duct, the airflow out of the lower rotor is compressed sharply and spreads rapidly along the ground. With the decrease in the distance between the duct and the wall, the reflection degree of the airflow from the wall surface will become greater and more airflow will no longer just rise along the wall, but will rise above the duct and flow into the duct from the formed source point.

The ground vortex of the duct with the corner effect is somewhat different from that with the ground effect. When the distance between the duct and the wall is the same, as the ducted fan is further from the ground, the degrees of the ground vortexes will decrease. This rule no longer applies when the distance between the duct and the wall changes.

In general, when the ducted fan operates with the corner effect, the air flows freely from the side away from the wall to the top of the duct, while the natural flow from the side close to the wall is reduced with the decreasing distance of the prototype from the wall. In this case, the flow is obviously not symmetrical. Compared with the ground effect or wall effect of the flow field, the corner effect has a more turbulent and complex flow field, and there is an obvious self-exhausting and self-intaking phenomenon, which also brings great challenges to the stable hover of the prototype.

### 2.2. Analysis of Aerodynamic Performance in Ground Effect and Wall Effect

According to the previous analysis, it is found that the aerodynamic characteristics of the prototype will change under the ground effect or wall effect compared with the unconstrained environment. When the UAV operates with the ground effect, the influence of aerodynamic interference on the prototype is mainly lift, while when the UAV operates with the wall effect, the influence of aerodynamic interference on the prototype is a tilting moment. In order to clarify the range of aerodynamic characteristics with two effects, a lift characteristic diagram of the prototype with the ground effect and a tilting moment characteristic diagram with the wall effect are given. This is also helpful to obtain the datasets needed for the neural network modeling. 

The dimensionless parameters such as lift coefficient, torque coefficient, and tilting moment coefficient [37] can be presented as follows:

(1) $$\left\{ \begin{array}{l} C_{T}=\frac{T}{\rho A\Omega^{2}R^{2}}\\ C_{Q}=\frac{Q}{\rho A\Omega^{2}R^{3}} \end{array}\right.$$

where *T* denotes the lift, and *Q* denotes the torque. The air density is *ρ* = 1.225 kg/m^3^, *A* = π*R*^2^ is the rotor area, and Ω is the rotor rotation speed.

**Figure 5:**
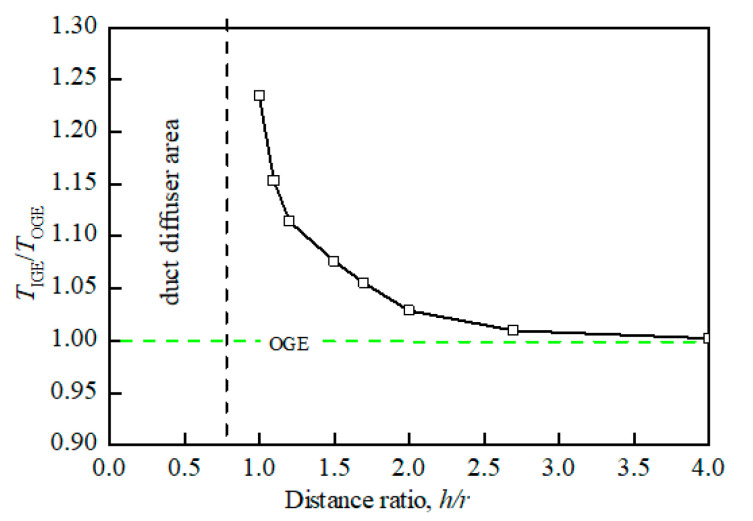
Connection diagram of lift ratio at different distance ratios (ground effect).

When the UAV with the ground effect, the main influence of the aerodynamic characteristics is reflected in the lift. The ground effect shown in Figure 3a will not only generate ground vortexes during the flow field analysis, but also increase the extra lift of the rotor at a constant power. It can be seen from Figure 5 that the shorter the distance from the ground is, the greater ratio *T*_IGE_/*T*_OGE_ of the prototype becomes, and the longer the distance from the ground is, the smaller ratio *T*_IGE_/*T*_OGE_ will be. Where *T*_IGE_ is the lift of the prototype hovering in an unconstrained environment, *T*_IGE_ is the lift of the prototype with ground effect. When the distance from the prototype to the ground exceeds 4r, the influences of ground effects can be ignored.

**Figure 6:**
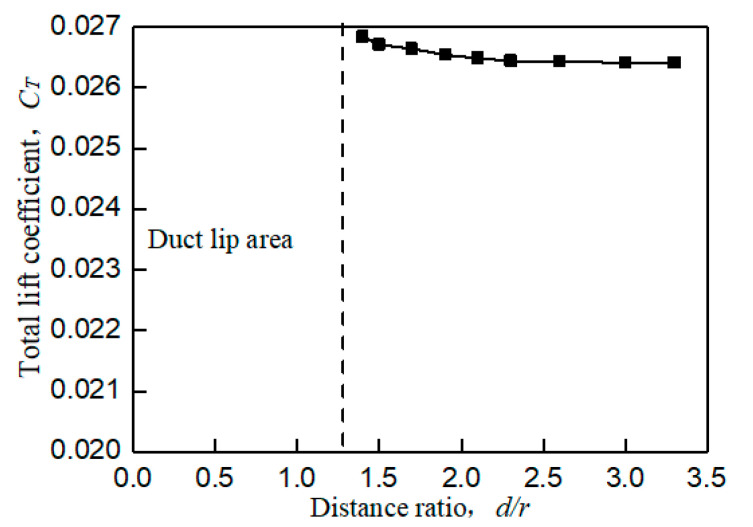
Connection diagram of total lift coefficient at different distance ratios (wall effect).

**Figure 7:**
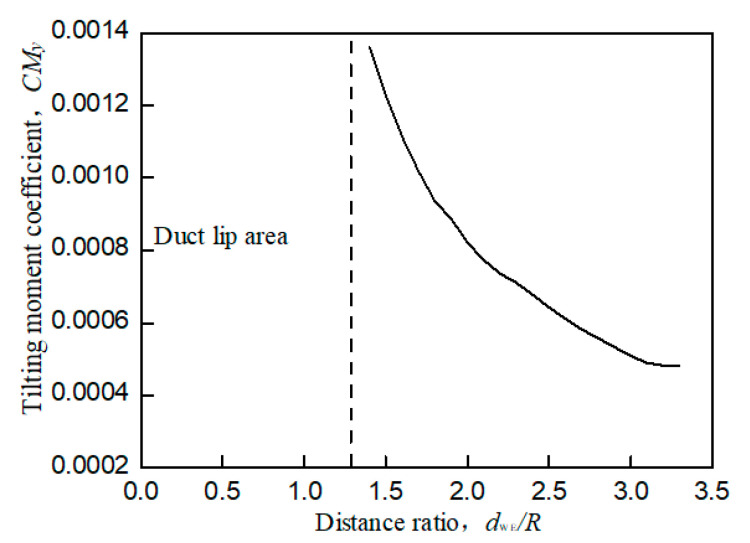
Tilting moment coefficient of wall effect by CFD simulations.

When the UAV with the wall effect, the main influence of the aerodynamic characteristics is focused on the tilt moment around the *y*-axis toward the wall. The wall effect shown in Figure 3b will produce unequal flow velocity and rate flowing into the duct lips on both sides during the flow field analysis, resulting in unbalanced force on the lip on both sides. This unbalanced force will generate a tilting moment *C_My_* that makes the prototype tilt toward the wall side, which is called a wall tilting moment. In addition, the wall effect will also have a slight impact on the lift of the prototype. When the prototype is closer to the wall, its total lift coefficient will also slightly increase, as shown in Figure 6. At the same time, as shown in Figure 7, the shorter the distance between the prototype and the wall is, the larger the tilting moment of the prototype becomes; the larger the distance between the prototype and the wall is, the smaller the tilting moment of the prototype becomes. When the distance between the prototype and the wall exceeds 3.8r, the influence of the wall effect can be ignored. 

**Figure 8:**
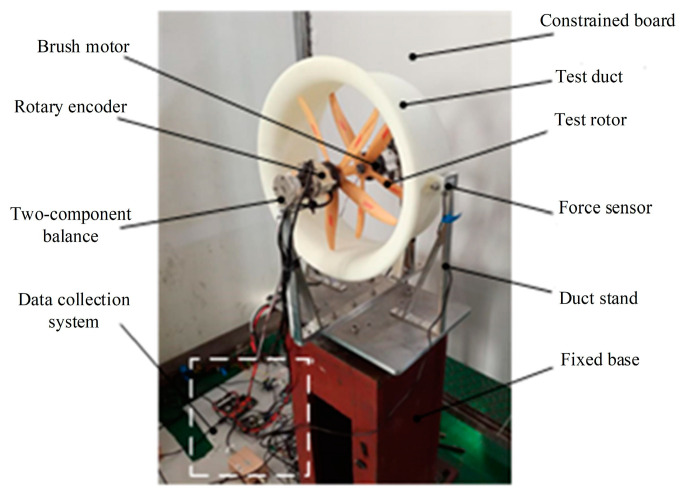
Ducted fan test bench.

Figure 5 and Figure 6 are both obtained from bench experiments. Their data were obtained by static testing after placing the constrained board in different positions and changing the distance from the duct. The test bench is shown in Figure 8; the lift of the duct was measured by the force sensor, and the force and torque of the rotor were measured by the two-component balance. All data in the experiment were stored and exported by the data collection system. In this paper, a constrained board was used instead of the ground or wall. In addition, because the test bench lacks moment equipment for measuring the X and Y directions of the ducted, the tilting moment cannot be obtained by the bench test. Therefore, Figure 7 is obtained by CFD simulation. 

**Figure 9:**
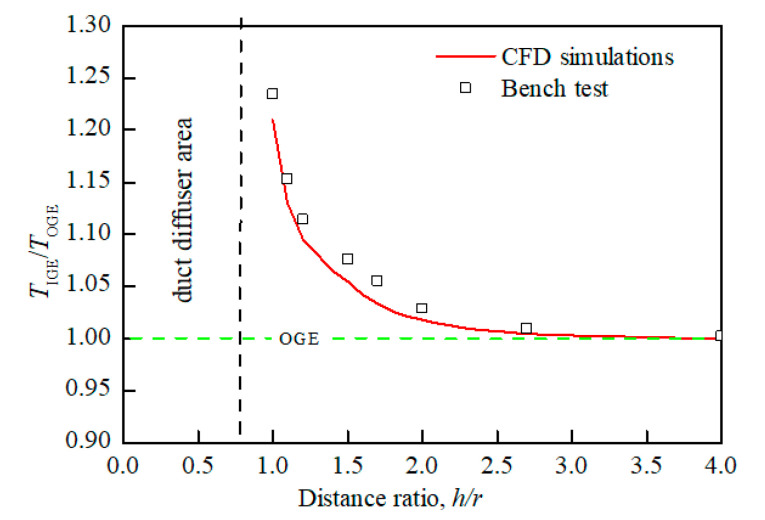
Lift ratio of ground effect by CFD simulations.

**Figure 10:**
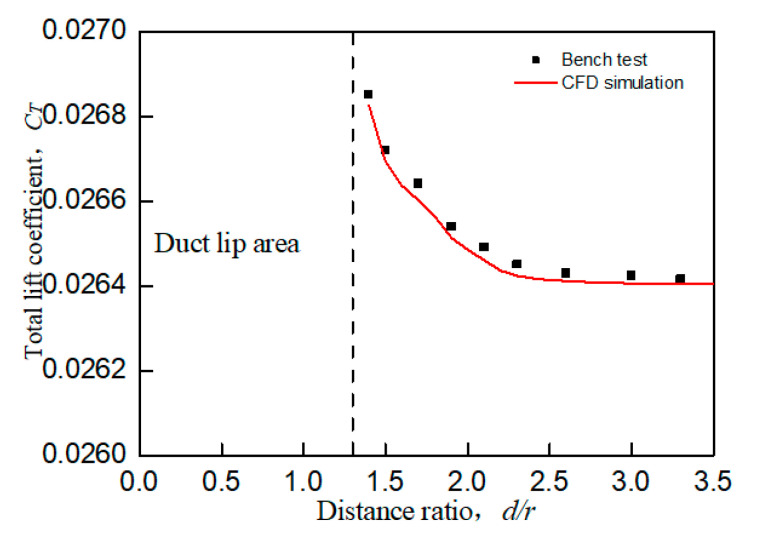
Total lift cofficient of wall effect by CFD simulations.

Figure 5 and Figure 6 illustrate a certain extent of the changes in the lift characteristics of the prototype as the distance changes. However, these two figures only reflect the changes in lift characteristics of an individual point at different positions and do not reflect the dynamic changes in lift characteristics with distance. In order to better explain the actual situation of lift characteristics changing with the distance, we need to obtain a continuous dynamic curve of lift characteristics changing with the distance. However, when the distance between the constrained board and the duct is dynamically adjusted, the external noise will increase, and the response of the sensor will be delayed. At the same time, the sensor response is very sensitive to the dynamic movement of the constrained board, which leads to large errors in the measured aerodynamic characteristics data. Therefore, similar to Figure 7, the CFD simulation was also used to set a smaller distance interval to describe the dynamic curve of lift characteristics with distance, as shown in Figure 9 and Figure 10.

Comparing Figure 5 with Figure 9 and Figure 6 with Figure 10, it is found that the trend of aerodynamic characteristics obtained by CFD simulation is consistent with the results of bench experiments. At the same time, it is found that the simulation results are close to the experiment results, which shows that the CFD simulation results can approximately fit the experimental results, indicating the rationality of the CFD simulation results. 

It can be seen from Figure 5, Figure 6, Figure 7, Figure 8, Figure 9 and Figure 10 that when the prototype operates with the ground effect or the wall effect, the aerodynamic characteristics of the prototype will change regularly as the distance from the prototype to the ground or wall changes. However, the aerodynamic characteristics of the prototype with the corner effect are variable and complicated, and do not change regularly with the distance. This is not similar to the ground effect or the wall effect, in that the change law cannot be obtained through a few studies at different positions. Therefore, it needs a neural network to model it.

## 3. Artificial Neural Network Models

Deep learning is a particularly popular research method nowadays. It can learn the internal laws and representation levels of sample data through multi-layer neural networks, and it has been widely used in many fields in recent years [38,39,40,41,42,43]. At the same time, neural networks are also considered as a useful method for unsteady aerodynamic modeling. When the prototype is flying in a corner environment, the force and moment of the prototype at each position cannot be obtained through CFD analysis or a bench test. At this time, the neural network is used to establish a reliable and accurate surrogate model, and the forces and moments of the prototype at different positions can be obtained according to a limited number of CFD analyses and experimental results. Once a network is trained, output generation takes mere seconds, which saves a lot of time compared to numerical simulation and experimentation. This is also of great convenience when designing the controller so that the prototype can hover and work stably in the corner environment.

In this section, we study the neural network architecture and training algorithms to obtain the optimal neural network model that is suitable for this paper. The rotor speed and the different distances of the prototype to the ground and the wall are inputs. At the same time, the forces and moments of the prototype in the three directions in the corner environment are outputs, respectively. All output data are organized as a dataset. This paper uses the hold-out method to randomly divide the dataset into two categories, namely training data and test data, with a ratio of 8:2. Since the hyperparameters of the neural network in this paper are optimized and determined by subsequent related algorithms, there are no validation data. The neural network training data in this paper are based on CFD numerical simulation results. The reason why the experimental data are not selected as the training dataset is that it takes a lot of manpower and time to obtain huge amounts of experimental data, and the experimental data contain noise, which causes uncertainty.

Therefore, six neural network models are built in this paper. This section takes the prototype lift as the output in the corner environment as an example, and other outputs of neural network models are built and trained similarly.

### 3.1. Neural Network Architectures

Before building a neural network model, we need to clarify the neural network structure to be established, such as the type of network, the architectures of network, input and output, etc.

Since this paper studies the force and moment of the prototype hovering in a corner environment at various positions, the state of the prototype is mainly hovering state and does not involve high-speed flight, so the state has nothing to do with time. Therefore, we can choose a back propagation (BP) artificial neural network.

A BP network which uses the gradient descent training algorithm to adjust its weights and biases is a multi-layer forward feedback network. It is composed of an input layer, an output layer and some hidden layers. The given input data are input from the input layer, and the hidden layer undergoes correlation transformation, and then the output data are obtained from the output layer.

The input and output of the neural network have been clearly given in Section 3. In addition, it is necessary to determine the number of layers and neurons and the number of hidden layers. It has been shown by studies that a S-type hidden layer plus a linear output layer of a three-layer BP network can map any function. Therefore, the number of hidden layers in this paper is determined to be one.

**Figure 11:**
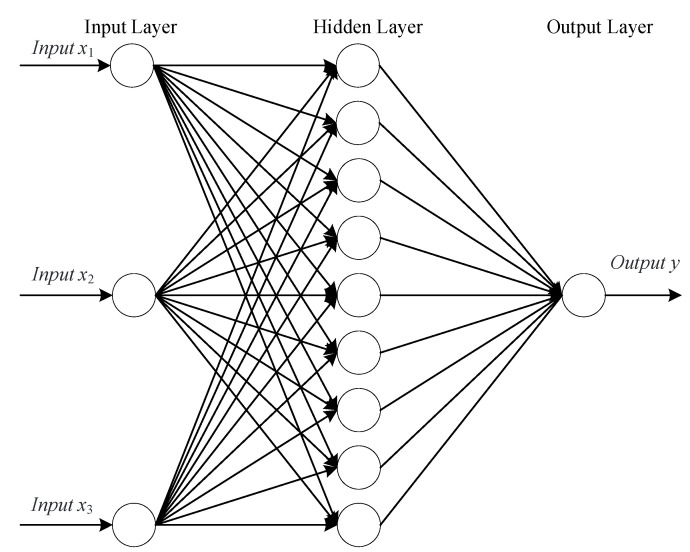
Back propagation neural network architectures.

Obtaining a reasonable number of neurons in the hidden layer is also a core step to establish a BP neural network, which will greatly affect the ability of the network to map complex problems. If the neuron number of the hidden layer is too low, the learning effect of the network will not be good, and training times need to be increased, and the accuracy of training will be affected; however, if the neuron number is too large, the training time will increase, and the network will be prone to overfitting. Generally speaking, the selection of the optimal neuron number in the hidden layer can be written as:

(2) $$b=\sqrt{a+c}+l$$

where *a*, *b*, and *c* denote the number of neurons in the input layer, hidden layer and output layer, respectively. *l* denotes a positive integer within 10. Through calculation, it is found that the value range of b is between 2 and 12. According to the trial and error method, it is found that when *b* = 9, the loss function is the smallest. Therefore, the structure of the network is finally chosen as three layers, and the number of neurons in the three layers is 3, 9, and 1, respectively. Figure 11 shows the architecture of the established BP artificial neural network.

In Figure 11, *x*_1_ is the distance from the prototype to the ground, *x*_2_ is the distance from the prototype to the wall, and *x*_3_ is the rotor speed. y is the lift of the prototype at different positions in a corner environment.

**Figure 12:**
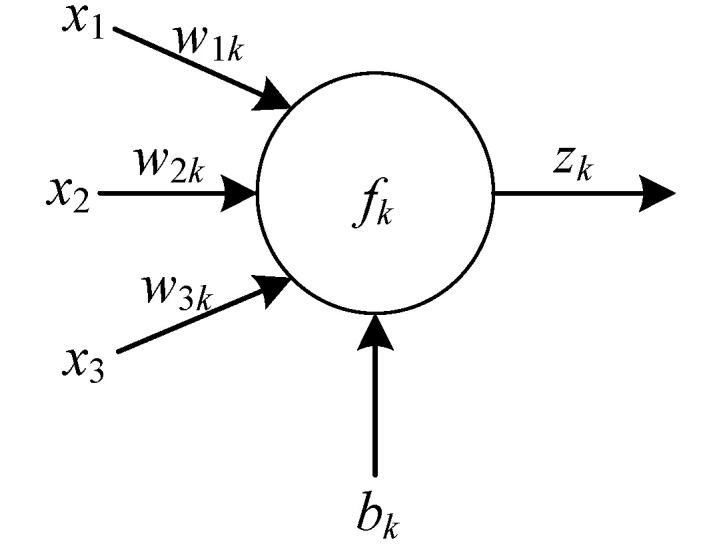
Working principle diagram of hidden layer neurons.

Figure 12 shows the working principle of each neuron in the hidden layer. Different inputs *x*_1_, *x*_2_, and *x*_3_ are weighted summation by corresponding connection weights $w_{ik}$ (*i* = 1, 2, 3) and inputted into each neuron in the hidden layer. Then neuronal bias *b_k_* is added to the neuronal weighted summation and the result **z***_k_* is obtained by mapping nonlinear activation function ***f****_k_*. The mapped signal is output by the neural network to obtain the final output result.

(3) $$z_{k}=f(x_{1}*w_{1k}+x_{2}*w_{2k}+x_{3}*w_{3k}+b_{k})$$

Similar to the process of obtaining z*_k_* by *x*_1_, *x*_2_ and *x*_3_, each **z***_k_* can be used as an input to obtain an output y through mapping.

### 3.2. Neural Network Training

For ensuring that a neural network has good modeling accuracy and fitting effect, the training algorithm needs to adjust its weights and thresholds constantly. The loss function of the training weights and biases is determined by the minimum square sum of the lift in the training dataset subtracted from the output lift of the neural network model.

(4) $$MSE=L_{\theta }=\frac{1}{N}\sum_{i=1}^{N}({\hat{y}}_{i}-y_{i}{)}^{2}$$

where *N* denotes the data number of the dataset, $\hat{y}$ is the output of the neural network and y is the actual output. θ denotes the vector of weights and biases of neural networks.

After determining the network structure and training dataset, the training algorithm can be set. The BP network generally uses the gradient descent algorithm to train the network. However, in practice, the gradient descent algorithm is not suitable, and there are many shortcomings, mainly the long time-consuming training, low iteration rate, and easy ability to make the weights converge to a local minimum, which lead to network training failure.

To solve the drawbacks of slow convergence and minimal localization of the gradient descent algorithm, an Adam (adaptive moment estimation) algorithm is chosen as the training algorithm for the neural network. The Adam algorithm is different from the gradient descent method in that it can adaptively adjust the learning rate of the neural network by calculating the first and second moment estimates of gradient. Steps (1)–(7) is the process of Adam algorithm to train neural network.

(1). *s* is the first order moment estimation and *q* is the second order moment estimation. Setting step size α, moment estimation attenuation rate $\lambda_{1}$, $\lambda_{2}$, initial parameters vector θ.
(2). Updating biased first moment estimate:
  (5) $$s\leftarrow \lambda_{1}s+(1-\lambda_{1})g$$
(3). Updating biased second moment estimate:
  (6) $$q\leftarrow \lambda_{2}q+(1-\lambda_{2})g⊙g$$
(4). Correcting the deviation of the first moment:
  (7) $$\hat{s}\leftarrow \frac{s}{1-\lambda_{1}{}^{t}}$$
(5). Correcting the deviation of the second moment:
  (8) $$\hat{q}\leftarrow \frac{q}{1-\lambda_{2}{}^{t}}$$
(6). Calculation update:
  (9) $$\Delta \theta =-\alpha \frac{\hat{s}}{\sqrt{\hat{q}}+\varepsilon }$$
(7). Using update:
  (10) θ←θ+Δθ
  where *t* is the current iterations, ε is a small constant, *g* is the gradients.
  (11) $$g=\frac{1}{N}\nabla_{\theta }\sum_{i=1}^{N}L_{i}(\theta )$$

### 3.3. Genetic Algorithm (GA) to Optimize Initial Weights of Neural Network Models

Generally, the initial weights of neural networks are randomly generated. Different initial weights will lead to different prediction performances of the network. If the initial connection weight is improperly selected, it will cause network oscillation, non-convergence or too long training time. Therefore, it is necessary to find a globally optimal solution of initial weights.

The genetic algorithm (GA) is an algorithm developed from biological evolution theory, which can successfully solve the global optimization problem. The genetic algorithm can not only design the neural network well, but also help to obtain the global optimal solution. Steps (1)–(7) is the process of GA optimization of the BP neural network.

(1). Encoding. Before using GA, it is necessary to encode the research object. The code length *L* can be written as:
  (12) L=a∗b+b∗c+b+c
(2). Getting the fitness function. After coding, the performance of different individuals needs to be evaluated according to the fitness value.
  (13) $$f=\frac{1}{MSE}$$
  where *f* is the fitness value.
(3). Select operation. Through selection operations, the next generation can inherit the optimized individual or obtain a new individual generated by cross pairing. After obtaining the fitness value, the roulette method can be used for individual selection. The probability of each individual *i* being selected can be given by the following Formula (13):
  (14) $$p_{i}=\frac{f_{i}}{\sum_{i=1}^{l}f_{i}}$$
  where *f_i_* denotes the fitness value of *i*-th individual, and *l* denotes the populations number.
(4). Cross operation. Cross operation is a key step of genetic algorithm. It can make two different chromosomes cross on one gene. The cross operation is shown in Equation (14).
  (15) $$\left\{ \begin{array}{l} g_{mi}=g_{mi}(1-a)+g_{mi}a\\ g_{ni}=g_{ni}(1-a)+g_{ni}a \end{array}\right.$$
  where *a* denotes a random number between 0 and 1, *g_mi_* denotes the *i*-th gene of the *m*-th chromosome, and the *i*-th gene of the *n*-th chromosome after the cross operation is expressed as *g_ni_*.
(5). Mutation operation. The gene value change on some chromosomes of the individual strings in the population can be realized by mutation operation, and the mutation operation can make the genetic algorithm have variability characteristics.

In order to better explain the principle of mutation, the *j*-th gene *c_ij_* of the *i*-th individual is selected as an example for mutation. The mutation process is shown in Equation (15).

(16) $$K=\left\{ \begin{array}{l} \frac{m_{1}(f_{\max}-f)}{f_{\max}-f_{avg}}\begin{array}{cc} & f>f_{avg} \end{array}\\ m_{2}\begin{array}{cccc} & & & \begin{array}{cc} & f<f_{avg} \end{array} \end{array} \end{array}\right.$$

where *f*_max_ denotes the population’ maximum fitness, *f*_avg_ denotes the population’ average fitness, *f* denotes the individual’ fitness, *m*_1_, *m*_2_ denotes the random number in the range of 0–1, and *K* denotes the mutation operator.

(6). The generation of new species. After mutation, inserting new individuals into the initial population to get a new population. Assigning the new individual weights to the neural network, and then calculate its fitness value. At this point, if the optimization requirements are met or the number of iteration steps reaches the limit, the next step is performed; otherwise, the genetic algorithm continues to run.
(7). Decoding to get the optimal initial weight. After obtaining the set performance index points or the maximum genetic generation number, the optimal individual in the final population is decoded to get the optimal results. If the algorithm iteratively satisfies the optimization requirements or the number of steps reaches the limit, the optimal individual in the final population can be decoded to get the optimal weights; otherwise, return to (2).

**Figure 13:**
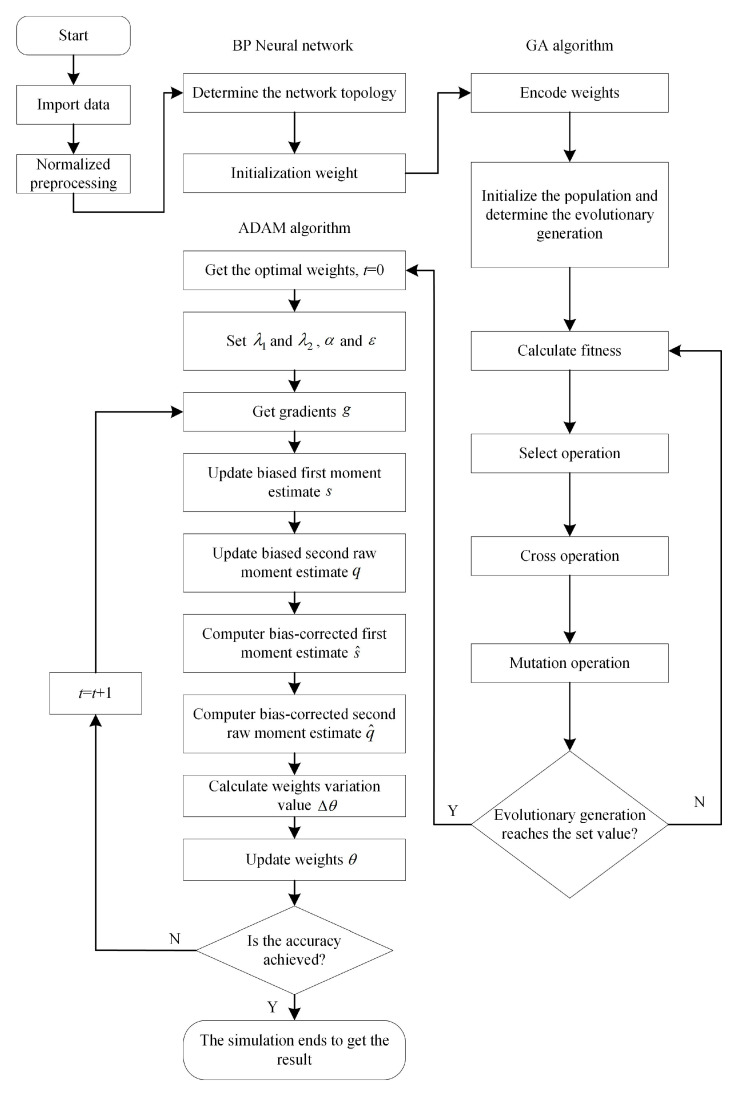
The process of applying genetic algorithm (GA) combined with Adam to BP neural network.

Figure 13 shows the process of applying GA combined with Adam to BP neural network.

## 4. Neural Network Models Simulation

**Figure 14:**
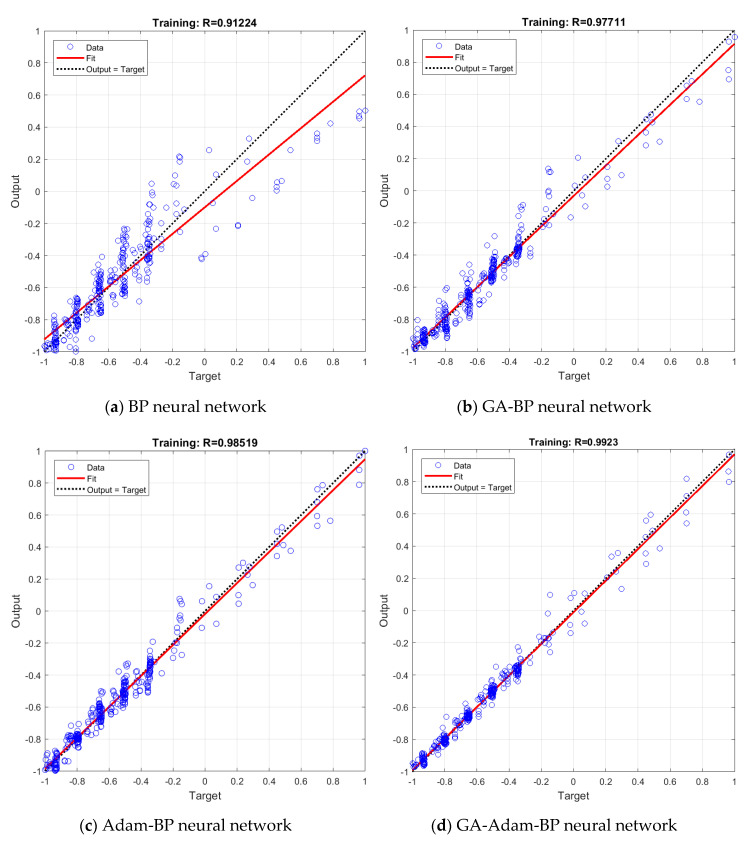
The regression analysis of four neural network models.

Through the combination of different training algorithms and genetic algorithms, four neural network models are obtained, the structures of which are BP, GA-BP, Adam-BP, and GA-Adam-BP. For these four models, the given three inputs are the same, and their outputs all are the predicted lift of the prototype. For the four kinds of neural networks, the same training dataset is selected, and through changing the training dataset, the model is trained a total of five times, and the one with the lowest cost function is selected as the final simulation result. Figure 14 shows the regression performance of the four neural network models.

**Figure 15:**
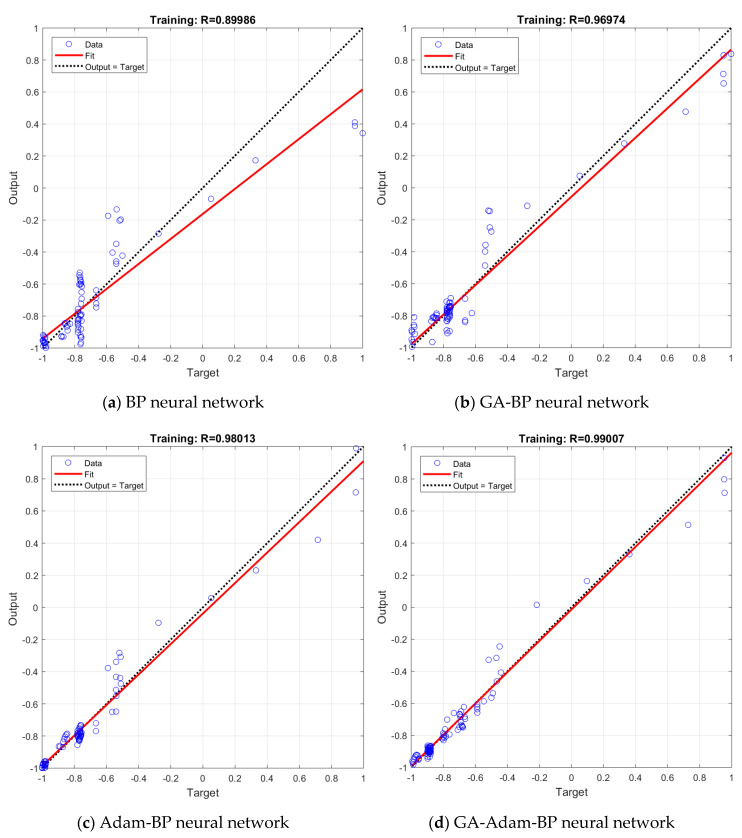
The regression analysis of test data of four neural network models.

In order to test the generalization ability of the established neural network models, the data in the dataset except for the training data are selected as the test dataset for the four models. The test dataset is substituted into the established models for training, and the corresponding training algorithm, network parameters and training process are the same as those described above. The test results of the four models after training are shown in Figure 15.

In Figure 14 and Figure 15, the output results of the neural network are drawn as several open circle points, the dotted line indicates the ideal fitting situation, and the solid line indicates the actual fitting accuracy. The regression performance of the neural network model can be expressed by the determination coefficient *R*^2^. The larger the value of *R*^2^ is, the higher the prediction accuracy of the model will be.

(17) $$R^{2}=1-\frac{\sum_{i=1}^{N}({\hat{y}}_{i}-y_{i}{)}^{2}}{\sum_{i=1}^{N}({\bar{y}}_{i}-y_{i}{)}^{2}}$$

where $\bar{y}$ denotes the average output, $\hat{y}$ denotes the predicted output, and y denotes desired output.

From the regression in Figure 14, we can obtain the determination coefficients *R*^2^ of the four neural network models as 0.8322, 0.9547, 0.9706, 0.9847, respectively. These values are all greater than 0.75, indicating that the mapping of three inputs to the output can be reflected by all four models effectively. The regression results of the four neural networks in Figure 14 show that the rank of the determination coefficients *R*^2^ from large to small is GA-Adam-BP>Adam-BP>GA-BP>BP; this shows that the GA-Adam-BP network model has the highest prediction accuracy and the determination coefficients *R*^2^ is very close to 1, while the BP network model has the lowest prediction accuracy. At the same time, compared with Figure 14a,b and Figure 14c,d, this shows that GA can effectively improve the prediction accuracy of the network in the case of the general performance of the network training algorithm, and when the performance of the training algorithm is good, its limited ability is improved. Figure 14b,c show that it is more valuable to find a more efficient train algorithm than to use GA to optimize the network.

From the regression in Figure 15, we can obtain the determination coefficients *R*^2^ of the four neural network models as 0.8097, 0.9404, 0.9607, 0.9802, respectively, and they are also greater than 0.75. Comparing Figure 14 with Figure 15, it can be seen that the determination coefficients of the test data and the training data of the four models are close, which indicates that the dataset is divided reasonably. At the same time, the rank of the determination coefficients *R*^2^ of test data from large to small is also GA-Adam-BP>Adam-BP>GA-BP>BP, indicating that the optimization algorithm can not only effectively improve the generalization ability of the neural network models, but also can be applied to improve the prediction accuracy of neural network models in different datasets.

**Figure 16:**
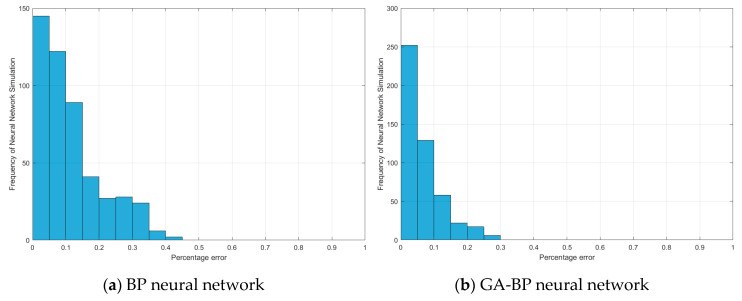

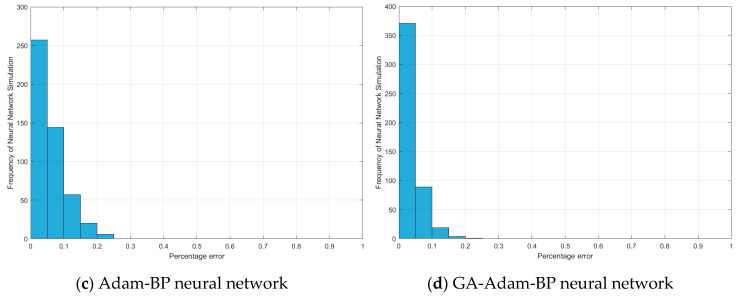
Histogram of percentage of four neural network models.

Although the regression performance chart in Figure 14 shows the fitting of the four models, it does not intuitively show the percentage of the different errors of each model in the training dataset. Figure 16 shows the number of data occupied by different percentage errors in the entire training dataset. The error distribution of most data of the four models is within 0–10%, and with the increase in the error percentage, the amount of data is generally decreased, which shows that the trained network model is reasonable. The more data there are in the lower percentage range, the better the fitting of the neural network is, which corresponds to the fitting accuracy of Figure 14.

*F*x, *F*y, *M*x, *M*y, and *M*z are used as the outputs of the other five neural network models, which are similar to the training, testing and building of the neural network model with lift as output. Due to the space limitations of this paper, the simulation diagrams of them are not given here. Table 2 shows the determination coefficients of the six neural network models.

According to the data in Table 1, in most cases, the determination coefficients *R*^2^ of the six neural network models are all greater than 0.75. The rank of the determination coefficients *R*^2^ of the other neural network models from large to small are GA-Adam-BP>Adam-BP>GA-BP>BP. Different neural network models use the same train algorithm to get different results due to their different training datasets and test datasets. With the optimization of the training algorithm and initial weights, the coefficient of determination of the training data gets closer and closer to 1, and the coefficient of determination of the test dataset is close to most of the training dataset, indicating that the neural network plays a good role in predicting the aerodynamic characteristics of the prototype under the corner effect.

## 5. Comparison of Different Surface Fitting Methods

**Figure 17:**
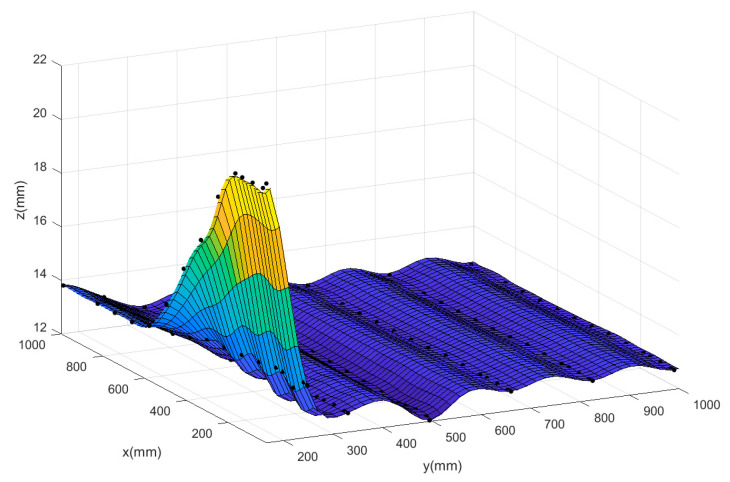
The predicted surface of the Kriging surface method.

**Figure 18:**
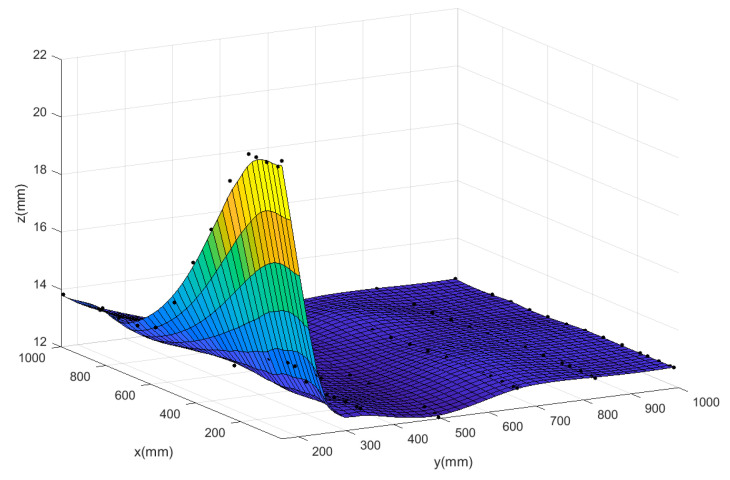
The predicted surface of the GA-Adam-BP neural network.

**Figure 19:**
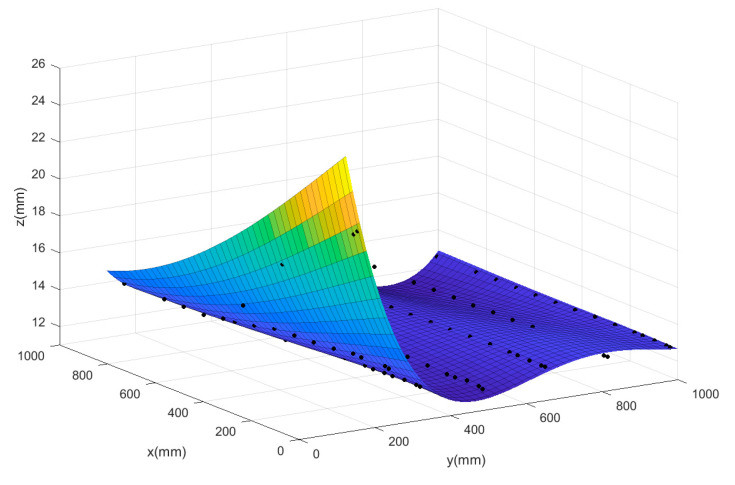
The predict surface of the polynomial fitting method.

To further illustrate that the neural network model is suitable for the comprehensive modeling of the prototype and the corner environment, it is necessary to use other data fitting methods to characterize the relationship between the lift of the prototype and its respective input parameters, and compare them with the neural network prediction model. Two different methods have been tested on the same CFD data to evaluate the quality of fitting: polynomial fitting and Kriging surface. This paper focuses on the effect of the relevant inputs of the corner environment on the force and moment of the prototype. Similar to the flow field analysis, taking the rotor speed at 4000 rpm as an example, a three-dimensional surface diagram of the total lift of the system and the distance from the prototype to the wall and the ground is made. The prediction surfaces of the three methods are shown in Figure 17, Figure 18 and Figure 19. The black scattered points in the figure represent the CFD lift simulation results when the ducted fan is at different positions, and the curved surface represents the predicted surface obtained by the various fitting methods.

Polynomial surface fitting uses a polynomial expansion to fit all points in an analysis area containing several data points to obtain the predicted distribution law of the data. The expansion coefficient is determined by least squares fitting, which means that the sum of squared errors between the calculated value and the true value should be minimized. Polynomial surface fitting based on least squares has been used in many fields due to its convenience and quick fitting. At the same time, although Kriging surface originated from mining and geostatistical applications, it also can be used for data fitting in practical aerospace applications [44].

The Mean Square Error (MSE) results of scattered data fitting by the three methods are shown in Table 3. According to the comparison of the surface graph in Figure 17, Figure 18 and Figure 19 and the results in Table 3, it is found that the polynomial fitting has the largest error, and the fitting accuracy is not high when there are many scattered data points; this indicates that the polynomial fitting method is not suitable for modeling the prototype combined with the corner environment. At the same time, it can be seen from the figure that using the autocorrelation and covariance methods, the kriging surface can accurately pass through most data points of model training. Therefore, the fitting error rate associated with the training dataset is very low. However, the disadvantage of this method is that the surface introduces artificial curvature between the original data points, and the resulting curved surface is not smooth enough, and the prediction surface fluctuates in a certain interval, making the predicted trend distorted and unreliable. Finally, although the MSE of the neural network prediction model is larger than that of the Kriging surface method, the neural network can produce a smooth surface to smooth any scattered points in the data. The neural network surface can make a good trade-off between MSE and smoothing rate so as to predict the trend of the whole region based on limited data points, which also shows the rationality of the neural network for modeling the prototype combined with the corner environment.

## 6. Conclusions

Sometimes the ducted fan aerial robot will hover near the corner, and the interference of the corner effect will prevent the prototype from working near the object in the corner environment. To clarify the unsteady aerodynamic characteristics of the prototype hovering in the corner effect, the flow field of the prototype in corner effect is analyzed at different positions. At the same time, six neural networks that combine the prototype with the corner environment are built, and the resultant forces and moments of the prototype hovering at different positions in the corner environment are obtained, respectively. The initial weights of the neural networks are optimized by GA, and then the neural network models are trained with gradient descent algorithms and Adam algorithms, respectively. The simulation results show that the BP neural network obtained by combining GA with the Adam training algorithm has better prediction accuracy. At the same time, by comparing the neural network with the polynomial fitting and the kriging surface method, it shows the validity of the neural network model for comprehensively modeling the prototype and the corner environment.

Further studies on the proposed neural network model need to be performed. For instance, if the ducted fan aerial robot needs to work in a more complex environment or the prototype works in a non-hovering state, the structures and optimization algorithms of the neural network models will be considered in more detail. In additional, the control for the prototype in complex environments on the basis of the neural network is also one of the focuses of future research.

## Figures and Tables

**Table 1:** Structural parameters of the prototype.

Parameter	Physical Description	Value
*m*_b_	Mass of the prototype	4.6 kg
*I*_xx_	Inertia tensor of the prototype around *x*-axis	0.092 kg·m^2^
*I*_yy_	Inertia tensor of the prototype around *y*-axis	0.283 kg·m^2^
*I*_zz_	Inertia tensor of the prototype around *z*-axis	0.245 kg·m^2^
*p*_cd_	Distance between duct center and C.G. of Prototype	0.32 m
*r*	Propeller radius	0.165 m
*n*_d_	Blade number of each disc	4
*c*	Blade chord length	0.027 m
*β*_0_	Attack angle at the root of blade	35°

**Table 2:** The modeling results of four methods for six aerodynamic parameters.

	*R* ^2^							
	BP		GA-BP		Adam-BP		GA-Adam-BP	
	Train	Test	Train	Test	Train	Test	Train	Test
*F*x	0.7351	0.7537	0.8976	0.9019	0.9256	0.9297	0.9378	0.9411
*F*y	0.7845	0.7668	0.9235	0.9071	0.9527	0.9403	0.9701	0.9613
*F*z	0.8322	0.8097	0.9547	0.9404	0.9706	0.9607	0.9847	0.9802
*M*x	0.8433	0.8482	0.9614	0.9601	0.9712	0.9722	0.9885	0.9840
*M*y	0.8256	0.8292	0.9511	0.9552	0.9680	0.9741	0.9788	0.9855
*M*z	0.7468	0.7504	0.9038	0.8947	0.9314	0.9325	0.9408	0.9354

**Table 3:** The Mean Square Error value of three methods.

The Fitting Method	MSE
Polynomial fitting	2.399 × 10^−1^
Kriging surface	1.256 × 10^−4^
Neural network	4.267 × 10^−3^
